# Inferring extinction in North American and Hawaiian birds in the presence of sighting uncertainty

**DOI:** 10.7717/peerj.2426

**Published:** 2016-09-01

**Authors:** David L. Roberts, Ivan Jarić

**Affiliations:** 1 Durrell Institute of Conservation and Ecology, School of Anthropology & Conservation, University of Kent, Canterbury, Kent, United Kingdom; 2 Leibniz-Institute of Freshwater Ecology and Inland Fisheries, Berlin, Germany; 3 Institute for Multidisciplinary Research, University of Belgrade, Belgrade, Serbia

**Keywords:** Avian extinction, Conservation triage, Critically endangered, Sighting records, Sighting reliability, Species persistence

## Abstract

For most species the timing of extinction events is uncertain, occurring sometime after the last sighting. However, the sightings themselves may also be uncertain. Recently a number of methods have been developed that incorporate sighting uncertainty in the inference of extinction based on a series of sightings. Here we estimate the timing of extinction for 41 of 52 North American and Hawaiian bird taxa and populations, the results of which suggest all became extinct before 2009. By acknowledging sighting uncertainty it results in two opposite effects, one pushing the timing of extinction away from the last sighting and the other drawing the timing of extinction nearer to it. However, for 14 assessed taxa and populations the upper 95% bounds lie beyond the end of the observation period and therefore suggest the possibility of continued persistence. This has important implications for conservation decision-makers and potentially reduces the likelihood of Romeo’s Error.

## Introduction

For many species our knowledge of their persistence is based on sightings that vary in quality and therefore the level of reliability (Roberts, Elphick & Reed, 2010). For species that are approaching extinction or that may already be extinct acknowledging this uncertainty can have profound effects on conservation decision-making, as erroneous evidence based on uncertain sightings can result in wasted resources (McKelvey et al., 2008). For example in 2005, based on a brief sighting and a pixelated image, the ivory-billed woodpecker was declared to have been rediscovered (Fitzpatrick et al., 2005), resulting in the mobilisation of resources for management strategies and recovery plans (Gotelli et al., 2012). However, based on the evidence its rediscovery was brought into question (Sibley et al., 2006), and subsequent extensive searches have failed to result in further sightings (Gotelli et al., 2012)

Several methods have been developed for the inference of extinction based on sighting data (see Solow, 2005 for a review), however until recently, these methods treated all sightings as certain. It has therefore been the responsibility of those using the methods to decide what data should be used and what should be discarded. Recently a number of methods have been developed that incorporate uncertainty (e.g., Solow et al., 2012; Jarić & Roberts, 2014; Lee et al., 2014).

Elphick, Roberts & Reed (2010) estimated the time of extinction for 38 of 52 North American and Hawaiian bird taxa and populations that are thought to be potentially extinct, along with the likelihood of extinction by 2009. In the study they based their analysis on sightings that are assumed to have the highest level of reliability (e.g., museum specimens), and then repeated the analysis by including additional sightings for which sufficient documentation exists to satisfy experts. In this way Elphick, Roberts & Reed (2010) attempted to acknowledge the issue of sighting uncertainty and incorporate it into their analysis on an *ad hoc* based criteria. Their analysis, however, excluded a number of controversial sightings that experts disagreed as to whether they should be accepted. In this paper we revisit this study, using a method that explicitly incorporates sighting uncertainty (Jarić & Roberts, 2014), to investigate the impact of accounting for sighting uncertainty when inferring extinction.

## Methods

We apply here the approach of Jarić & Roberts (2014) that represents a modification of the existing methods for inferring extinction based on sighting records, which allows for inclusion of specific sighting reliabilities of individual observations. In line with the original approach, we apply it to the standard Solow method (Solow, 1993), which was also used to infer extinction by Elphick, Roberts & Reed (2010). For details on Solow method modification, see Jarić & Roberts (2014) as well as Supplemental Information 1.

We revisited the 52 North American bird taxa and populations assessed by Elphick, Roberts & Reed (2010) that are presumed to be extinct, or whose persistence is a point of discussion. In their study and used here, Elphick, Roberts & Reed (2010—supplementary material) compiled sighting records for all taxa but divided the sightings into three categories that form a nest hierarchy:

1. Physical Evidence (PE)—e.g., museum specimens, but also uncontroversial photographs, video, and sound recordings.
2. Independent Expert Opinion (IEO)—evidence that experts deemed sufficiently documented to confirm the record.
3. Controversial sightings (CS)—sightings judged to lack firm evidence including any sighting for which there is published disagreement between experts.

Elphick, Roberts & Reed (2010) used the method of Solow (1993) for the inference of extinction (but also see Solow, 2005) and based their analysis on PE and PE + IEO, but excluded CS. Following Jarić & Roberts (2014), who applied the sighting reliability scoring system used by BirdLife International (Table 1 of Lee et al., 2014), we assign PE sightings (i.e., Lee et al.’s “Record described as being based on collected individual”) with a lower limit of reliability of 0.8, and upper limit of 0.9 and a mean of 0.85. This was repeated for IEO (i.e., Lee et al.’s “Record based on observation described in the literature as ‘confirmed’ or considered fairly convincing”) and CS (i.e., Lee et al.’s “Record described in the literature as (or judged to be) unconfirmed or questionable”), 0.6, 0.8, 0.7 and 0.1, 0.4, 0.25 respectively.

First sightings in each sighting record dataset were used to establish the beginning of the sighting period, and excluded from the analysis (Solow, 2005). Minimum number of sightings in a sighting record (*n* ≥ 5, i.e., 4 following the exclusion of the first sighting) was defined in line with Solow (2005) and Elphick, Roberts & Reed (2010). Consequently, analyses were conducted only for sighting records and reliability score setups with the most likely number of observations (*r* value, see Jarić & Roberts, 2014) of at least 3.5 (i.e., excluding the reliability score for the first sighting). The approach was used to estimate the *p* value for each species (with *T* = 2009 in line with Elphick, Roberts & Reed, 2010), probable extinction time (*T*_*E*_) and the upper bound (*T*_*CI*_) of a 1-*α* confidence interval (*α* = 0.05).

## Results

Of the 52 taxa and populations, there were sufficient sightings to conduct analyses for 41, compared with 38 taxa and populations analyzed by Elphick, Roberts & Reed (2010). Estimated extinction dates (*T*_*E*_) ranged from 1855 to 2008, with the upper 95% bounds (*T*_*CI*_) on these estimates ranging from 1863 to 2113 (Table 1). Based on these analyses, there is no indication that any taxa and populations are likely to persist, including the ‘Alalā (Hawaiian crow, *Corvus hawaiiensis*) which was the only taxa in Elphick, Roberts & Reed’s (2010) study for which there was any indication of likely persistence. Taxa and populations for which the 95% confidence interval around the predicted extinction date includes dates after 2008 were Eskimo Curlew (*Numenius borealis*), Ivory-billed woodpecker (*Campephilus principalis*), ‘Alalā (Hawaiian crow), Kaua‘i ‘ō‘ō (*Moho braccatus*), O‘ahu ‘ō‘ō (*M. apicalis*), Kama‘o (*Myadestes myadestinus*), Oloma‘o (Moloka‘i) (*M. lanaiensis rutha*), ‘Ō‘ū (Kaua‘i) (*Psittirostra psittacea*), Nukupu‘u (Kaua‘i) (*Hemignathus lucidus hanapepe*), Nukupu‘u (Maui) (*H. l. affinis*), O‘ahu ‘alauahio (*Paroreomyza maculata*), Maui ‘akepa (*Loxops coccineus ochraceus*), Oahu ‘akepa (*L. c. rufus*) and the Po‘o-uli (*Melamprosops phaeosoma*) (indicated in bold in Table 1). In comparison, Elphick, Roberts & Reed’s (2010) analysis only observed such confidence intervals for the ‘Alalā (Hawaiian crow), as well as partly for Kama‘o, O‘ahu ‘alauahio and the Po‘o-uli (i.e., they had *T*_*CI*_ > 2009 only when using PE, while for PE + IEO combination it was *T*_*CI*_ < 2009). Elphick, Roberts & Reed (2010) only provided sighting data to 2009, and therefore other, most likely controversial, sightings may have occurred during the following years, assuming no further sightings have actually occurred since 2009. Taxa and populations for which the 95% confidence intervals around the predicted extinction dates include dates after 2016 were ‘Alalā (Hawaiian crow), Oloma‘o (Moloka‘i), Nukupu‘u (Kaua‘i), Nukupu‘u (Maui), O‘ahu ‘alauahio, Maui ‘akepa and the Oahu ‘akepa (Table 1).

**Table 1:** Evaluated North American and Hawaiian bird taxa potentially considered extinct. IUCN Red List category (http://www.birdlife.org/datazone/species accessed July 2016; CR(PE), Critically Endangered (Possibly Extinct); EW, Extinct in the Wild; EX, Extinct), year of last reported sighting including controversial sightings reported up to 2009 (Elphick, Roberts & Reed, 2010—supplementary material), number of years with confirmed records (*n*). Sighting reliability estimates give the upper, mean and lower sighting reliabilities as described in the methods. *p* is the probability of a sighting record in 2009, *T*_*E*_ estimated year of extinction, and *T*_*CI*_ the upper 95% bound on that estimate of *T*_*E*_. Years highlighted in bold represent results that do not support extinction.

Species	IUCN Red List	Last sighting	*n*	Sighting reliability	*p*	*T*_*E*_	*T*_*CI*_
Labrador duck (*Camptorhynchus labradorius*)	EX	1878	13	Upper	3E−7	1880	1889
				Mean	7E−7	1879	1889
				Lower	2E−6	1879	1890
Heath hen (*Tympanuchus c. cupido*)	EX	1932	39	Upper	4E−15	1933	1936
				Mean	7E−14	1933	1936
				Lower	1E−12	1933	1936
Laysan rail (*Zapornia palmeri*)	EX	1945	29	Upper	7E−9	1946	1951
				Mean	3E−8	1946	1952
				Lower	1E−7	1946	1952
Hawaiian rail (*Zapornia sandwichensis*)	EX	1893	9	Upper	0.010	1905	1956
				Mean	0.014	1903	1961
				Lower	0.018	1900	1965
Eskimo curlew (*Numenius borealis*)	CR(PE)	2006	49	Upper	0.062	2003	**2010**
				Mean	0.028	1999	2007
				Lower	0.004	1989	1997
Great auk (*Pinguinus impennis*)	EX	1888	24	Upper	8E−10	1872	1879
				Mean	1E−9	1865	1872
				Lower	1E−9	1855	1863
Passenger pigeon (*Ectopistes migratorius*)	EX	1907	26	Upper	3E−15	1906	1909
				Mean	2E−14	1905	1908
				Lower	8E−14	1904	1907
Carolina parakeet (*Conuropsis carolinensis*)	EX	1950	50	Upper	1E−10	1946	1950
				Mean	4E−10	1942	1947
				Lower	3E−10	1933	1938
Ivory-billed woodpecker (*Campephilus principalis*)	CR	2006	68	Upper	0.065	2005	**2010**
				Mean	0.019	2000	2006
				Lower	5E−4	1987	1993
‘Alalā (Hawaiian crow) (*Corvus hawaiiensis*)	EW	2003	68	Upper	0.220	2007	**2015**
				Mean	0.251	2007	**2017**
				Lower	0.286	2008	**2018**
Kaua’i ‘ō‘ō (*Moho braccatus*)	EX	2001	43	Upper	0.103	2002	**2013**
				Mean	0.080	2000	**2012**
				Lower	0.055	1996	**2010**
O‘ahu ‘ō‘ō (*Moho apicalis*)	EX	1976	10	Upper	0.292	1994	**2113**
				Mean	–	–	–
				Lower	–	–	–
Bishop’s ‘ō‘ō(Moloka‘i) (*Moho bishopi*)	EX	1904	5	Upper	3E−4	1907	1919
				Mean	–	–	–
				Lower	–	–	–
Hawai‘i ‘ō‘ō (*Moho nobilis*)	EX	1976	24	Upper	0.008	1974	1991
				Mean	0.006	1967	1985
				Lower	0.001	1944	1963
San Clemente [Bewick’s] wren (*Thryomanes bewickii leucophrys*)	–	1941	20	Upper	7E−7	1944	1951
				Mean	1E−6	1944	1951
				Lower	3E−6	1944	1952
Laysan millerbird (*Acrocephalus f. familiaris*)	EX	1916	12	Upper	2E−6	1919	1927
				Mean	4E−6	1919	1928
				Lower	9E−6	1919	1929
Kama‘o (*Myadestes myadestinus*)	EX	1999	50	Upper	0.069	2001	**2011**
				Mean	0.067	2000	**2011**
				Lower	0.050	1997	**2009**
Oloma‘o (Moloka‘i) (*Myadestes lanaiensis rutha*)	CR(PE)	2005	16	Upper	0.188	2001	**2025**
				Mean	0.154	1998	**2024**
				Lower	0.129	1993	**2024**
Oloma‘o (Lāna‘i) (*Myadestes l. lanaiensis*)	CR(PE)	1934	9	Upper	0.001	1941	1960
				Mean	0.003	1941	1963
				Lower	0.005	1942	1967
Bachman’s warbler (*Vermivora bachmanii*)	CR(PE)	2001	61	Upper	0.004	1997	2002
				Mean	0.001	1993	1998
				Lower	4E−5	1981	1987
Dusky seaside sparrow (*Ammodramus maritimus nigrescens*)	EX	1980	48	Upper	6E−5	1983	1988
				Mean	1E−4	1983	1989
				Lower	2E−4	1983	1989
‘Ō‘ū (Kaua‘i) (*Psittirostra psittacea*)	CR(PE)	1997	33	Upper	0.057	2000	**2010**
				Mean	0.060	1999	**2010**
				Lower	0.055	1996	**2010**
‘Ō‘ū (Hawai‘i) (*Psittirostra psittacea*)	CR(PE)	1987	42	Upper	0.004	1990	1998
				Mean	0.007	1991	2000
				Lower	0.013	1991	2001
‘Ō‘ū (Moloka‘i) (*Psittirostra psittacea*)	CR(PE)	1965	6	Upper	0.015	1940	1978
				Mean	0.010	1929	1964
				Lower	–	–	–
‘Ō‘ū (Lāna‘i) (*Psittirostra psittacea*)	CR(PE)	1927	8	Upper	9E−4	1933	1951
				Mean	0.001	1933	1953
				Lower	0.002	1933	1955
‘Ō‘ū (Maui) (*Psittirostra psittacea*)	CR(PE)	1945	7	Upper	0.004	1927	1954
				Mean	0.004	1919	1947
				Lower	0.003	1911	1938
Greater koa-finch (*Rhodacanthis palmeri*)	EX	1967	8	Upper	0.007	1943	1970
				Mean	0.003	1928	1952
				Lower	7E−4	1911	1927
Greater ‘amakihi (*Hemignathus sagittirostris*)	EX	1901	5	Upper	9E−5	1903	1912
				Mean	–	–	–
				Lower	–	–	–
Lesser ‘akialoa (*Hemignathus obscurus*)	EX	1940	19	Upper	5E−6	1923	1934
				Mean	4E−6	1917	1928
				Lower	3E−6	1911	1923
Greater ‘akialoa (Kaua‘i) (*Hemignathus ellisianus stejnegeri*)	EX	1995	21	Upper	0.027	1991	2004
				Mean	0.016	1985	2000
				Lower	0.009	1978	1994
Nukupu‘u (Kaua‘i) (*Hemignathus lucidus hanapepe*)	CR(PE)	1996	24	Upper	0.179	2002	**2022**
				Mean	0.198	2001	**2028**
				Lower	0.083	1983	**2019**
Nukupu‘u (Maui) (*Hemignathus lucidus affinis*)	CR(PE)	1996	24	Upper	0.256	2004	**2029**
				Mean	0.346	2007	**2047**
				Lower	0.322	2001	**2086**
O‘ahu ‘alauahio (*Paroreomyza maculata*)	CR(PE)	2002	46	Upper	0.218	2006	**2019**
				Mean	0.191	2004	**2020**
				Lower	0.099	1995	**2016**
Maui ‘alauahio (Lāna‘i) (*Paroreomyza montana*)	EX	1937	10	Upper	7E−4	1942	1958
				Mean	0.001	1942	1960
				Lower	0.002	1942	1961
Kākāwahie (*Paroreomyza flammea*)	EX	1963	16	Upper	0.006	1970	1987
				Mean	0.008	1969	1988
				Lower	0.009	1968	1989
Maui ‘akepa (*Loxops coccineus ochraceus*)	EX	1995	21	Upper	0.147	2001	**2019**
				Mean	0.144	1999	**2021**
				Lower	0.122	1995	**2021**
Oahu ‘akepa (*Loxops coccineus rufus*)	EX	1976	7	Upper	0.125	1965	**2053**
				Mean	0.097	1950	**2044**
				Lower	–	–	–
Hawai‘i mamo (*Drepanis pacifica*)	EX	1960	12	Upper	0.033	1943	1996
				Mean	0.035	1935	1996
				Lower	0.041	1926	2000
Black mamo (*Drepanis funerea*)	EX	1955	6	Upper	0.024	1944	1987
				Mean	–	–	–
				Lower	–	–	–
Laysan honeycreeper [‘apapane] (*Himatione sanguinea freethii*)	EX	1923	14	Upper	6E−4	1930	1950
				Mean	9E−4	1930	1952
				Lower	0.001	1929	1954
Po‘o-uli (*Melamprosops phaeosoma*)	CR(PE)	2004	27	Upper	0.037	2005	**2009**
				Mean	0.050	2005	**2009**
				Lower	0.068	2005	**2010**

## Discussion

Incorporating uncertainty in the inference of extinction of a species has two effects that run counter to each other, one potentially pushing forward the date of extinction and the other drawing it to an earlier year. Firstly, by reducing the reliability from 1.0 it increases uncertainty in the date of extinction and therefore results in the inferred persistence of the taxa being potentially pushed beyond those inferred through methods that do not incorporate uncertainty. Secondly, however, by allowing for the incorporation of uncertainty it is possible to incorporate controversial sightings (i.e., Elphick, Roberts & Reed, 2010 only incorporate PE and IEO). This results in more sightings within a record and therefore fewer gaps between years in the sighting record, thus potentially drawing the extinction date closer to the time of the last sighting, although the date of the last sighting is by definition uncertain (see Jarić & Roberts, 2014).

In this study, by incorporating sighting uncertainty into the inference of extinction it allowed us to assess an additional 3 taxa and populations beyond Elphick, Roberts & Reed’s (2010) 38, due to the additional data this brings from the controversial sightings. Furthermore, the number of taxa and populations for which the 95% confidence interval around the predicted extinction date includes dates after 2008 increased from 6 to 14. This has potentially important implications in terms of conservation management and the distribution of resources for the additional 8 taxa and populations. Further, improper classification of these taxa could have resulted in Romeo’s Error (Collar, 1998), where the taxon is assumed to be extinct, which results in a lack of appropriate and timely conservation efforts, and consequently precipitates its true extinction.

Sighting observations of species or individuals are likely to have some level of uncertainty as to whether a correct identification has been made. Few have, however, attempted to quantify the level of uncertainty (e.g., Lee et al., 2015), test for the level of accuracy experimentally (e.g., Gibbon, Bindemann & Roberts, 2015) or incorporated this into their analyses (e.g., Jarić & Roberts, 2014; Lee et al., 2014). As we have shown here, acknowledging such uncertainties can have a profound impact on decision-making; in the case of a critically endangered species, it may influence whether it is considered extinct or extant and therefore whether conservation efforts and resources should be allocated. For some species, extinction may occur within years of being described as a new taxon to science. As an example, a cryptically coloured treehunter from Brazil, *Cichlocolaptes mazarbarnetii*, described in 2014, was last seen in 2007, but had lain misidentified in the National Museum of Brazil for over 20 years having been collected in 1986 (Lees & Pimm, 2015).

Finally, while we incorporated sighting uncertainty into a time-based extinction model, such sightings with spatial data are frequently used in occupancy modelling with apparently little consideration to the underlying uncertainty of the identification (but see Romero et al., 2014). This is likely to be particularly an issue when using historic sightings, whose location data may also be imprecise. Much of this data is becoming increasingly available online and can be accessed rapidly. However, consideration should be given to the quality of the data, including spatial and temporal inaccuracies (Yesson et al., 2007), particularly identification uncertainties.

##  Supplemental Information

10.7717/peerj.2426/supp-1 Supplemental Information 1

## Additional Information and Declarations

### Data Availability

The following information was supplied regarding data availability: The data was previously published in Elphick CS, Roberts DL, Reed JM. 2010. Estimated dates of recent extinctions for North American and Hawaiian birds. Biological Conservation 143(3): 617–624.
