# Supplementary material for: Inferring extinction in North American and Hawaiian birds in the presence of sighting uncertainty
Source: PeerJ. 2016 Sep 1;4:e2426. doi: 10.7717/peerj.2426 (PMC5012411; doi:10.7717/peerj.2426)
Supplement: Supplemental Information 1 [file peerj-04-2426-s001.doc]

**Supplementary Information 1 for “Inferring extinction in North American birds in the presence of sighting uncertainty” (Roberts & Jarić)**

**Method description**

We present here the method by Jarić & Roberts (2014) that allows for inclusion of specific sighting reliabilities of individual observations when inferring extinction, and apply it to the Solow equation (Solow 1993):

$p\left(T_{n}\leq t_{n}\text{|}T_{E}=T\right)=\left(\frac{t_{n}}{T}\right)^{n}$ (1)

where *n* represents the number of time units in which the species was recorded within the whole sighting period (*T*), *T_E_* represents the time of extinction and *t_n_* is the time of the last positive sighting within the observation period, with the sighting records ordered from the earliest to the latest, *t*_1_ < *t*_2_ < … < *t_n_*.

The approach by Jarić & Roberts (2014) modifies the use of sightings as a binary sequence of presences and absences, so that the species presences are expressed as probabilities that represent reliability of each observation. If the reliability of the sighting record is not certain, a probability value can be assigned to each of the sightings (i.e., *p*_1_, *p*_2_, ... *p_n_*), indicating the likelihood that the given observation is true. For example, probability value close to 1 would correspond to a certain observation (e.g. specimens from museum collections), while values close to 0 would be assigned to observations likely to be false. Consequently, the total number of sightings (*n*) in Eq. 1 can be replaced by an index value, which represents a sum of probability values assigned to each of the observations in a sighting record:

$r=\sum_{i=1}^{n} p_{i}$ (2)

The value of *r* represents the most likely number of observations. Note that when all observations within a sighting record are certain, *r* will be equal to *n*, and the mean observation reliability is represented by *r* / *n*. If the last observation (*t_n_*) is likely to be false, the previous one (*t_n-_*_1_) should then be considered as the end of the sighting record. The probability that an observation is the last one in a sighting record is equal to the probability that it is a true observation (*p_i_*), multiplied with the probability that each of the consequent observations are false (1 - *p_i_*):

$p_{i}\times\prod_{a=i+1}^{n} \left(1-p_{a}\right)$ (3)

To account for this problem, the last observation (*t_n_*) in Eq. 1 should be replaced with *t_r_*, which represents the most likely endpoint of a sighting record:

$t_{r}=\sum_{i=1}^{n} \left(t_{i}\times p_{i}\times\prod_{a=i+1}^{n} \left(1-p_{a}\right)\right)$ (4)

Note that when the reliability of the last observation is equal to 1, *t_r_* is equal to *t_n_*. The modified Solow (1993) equation will then be:

$p=\left(\frac{t_{r}}{T}\right)^{r}$ (5)

$p=\left[\sum_{i=1}^{n} \left(t_{i}\times p_{i}\times\prod_{a=i+1}^{n} \left(1-p_{a}\right)\right)/T\right]^{\sum_{i=1}^{n} p_{i}}$ (6)

The same modification can be applied to the equations of Solow (1993) which estimate a uniform minimum variance unbiased estimate of the extinction time (*T_E_*) and the upper bound (*T_CI_*) of a 1-*α* confidence interval for *T_E_* (Solow 2005):

$T_{E}=\frac{r+1}{r}t_{r}$ (7)

$T_{CI}=\frac{t_{r}}{\alpha^{\left(1/r\right)}}$ (8)

For further information on method description, see Jarić & Roberts (2014).

**References**

Jarić, I., & Roberts, D. L. (2014). Accounting for observation reliability when inferring extinction based on sighting records. Biodiversity and Conservation, 23(11), 2801-2815.

Solow, A. R. (1993). Inferring extinction from sighting data. Ecology, 74, 962-964.

Solow, A. R. (2005). Inferring threat from a sighting record. Mathematical Biosciences, 195, 47-55.
